# Treatment outcomes of Pumani bubble-CPAP versus oxygen therapy among preterm babies presenting with respiratory distress at a tertiary hospital in Tanzania—Randomised trial

**DOI:** 10.1371/journal.pone.0235031

**Published:** 2020-06-30

**Authors:** Annette Baine Mwatha, Michael Mahande, Raimos Olomi, Beatrice John, Rune Philemon

**Affiliations:** 1 Kilimanjaro Christian Medical University College, Moshi, Tanzania; 2 Department of Pediatrics and Child Health, Kilimanjaro Christian Medical Center (KCMC), Moshi, Tanzania; 3 Gertrude`s Children`s Hospital, Nairobi, Kenya; 4 Institute of Public Health, Department of Epidemiology & Biostatistics, Kilimanjaro Christian Medical University College (KCMUCo), Moshi, Tanzania; RCSI & UCD Malaysia Campus (formerly Penang Medical College), MALAYSIA

## Abstract

**Background:**

Respiratory distress syndrome (RDS) is the most common respiratory disease in premature babies and the major cause of morbidity and mortality among preterm babies. Effective treatment of these babies requires exogenous surfactant and/or mechanical ventilation but these are of limited availability in low and middle income countries. A cheaper, simpler and more accessible treatment for preterms with RDS called bubble-continuous positive airway pressure (bCPAP) has been reported to be effective in treating RDS in preterm babies with varying levels of effectiveness ranging from 42% to 85%. We aimed to implement and determine the efficacy of bCPAP and its immediate outcomes as compared to oxygen therapy in preterm babies presenting with respiratory distress at a tertiary hospital in Tanzania.

**Method:**

A randomized control trial, conducted from December 2016 to May 2017, included all preterm babies admitted at the neonatal care unit presenting with signs of respiratory distress and meeting the inclusion criteria. The primary outcome was survival while the secondary outcomes were treatment duration, duration of hospital stay and treatment complications.

**Results:**

A total of 824 babies were admitted in the neonatal care unit during the study period. Of these, 187 babies were preterm and 48 babies were recruited and randomized (25 bCPAP vs 23 oxygen). The overall survival to discharge for all eligible participants (n = 48) was 58.2% compared to those who adhered to treatment protocol (n = 45, 62.2%). Babies in the bCPAP group had higher survival (17/22; 77.3%) as compared to their counterparts in the oxygen therapy group (11/23; 47.8%). Babies treated with bCPAP had 52% lower risk of death (crude HR 0.48, 95% CI = 0.16–1.43) compared to babies receiving oxygen therapy. The median duration of treatment for babies in the oxygen therapy group was 2 (Range 0–16) days compared to 2 (Range 0–5) days in the bCPAP group. The median duration of hospital stay for babies receiving bCPAP was 14 (range 7–43) days. Nasal bleeding was commonly observed among babies in the bCPAP group as compared to those in the oxygen therapy group.

**Conclusion:**

This study revealed that treatment with bCPAP had a 30% clinical improvement in survival to discharge. Our findings highlight the role of bCPAP in reducing neonatal mortality in resource limited settings but further adequately powered studies in this or similar settings are required.

## Introduction

Respiratory distress syndrome (RDS), also known as hyaline membrane disease, is the most common respiratory disorder in preterm babies. RDS is primarily caused by deficiency of pulmonary surfactant which is a surface active phospholipoprotein produced by alveolar Type 2 cells that helps prevent alveolar collapse by decreasing surface tension within the alveoli. RDS is the major cause of morbidity and mortality in preterm babies < 37 weeks of gestation age [1, 2]. According to the 2015 global countdown report, preterm deaths accounted for 18% of under-five mortality making it the leading cause of neonatal and under-five mortality [3]. In Tanzania, preterm mortality accounts for 10% of the neonatal deaths and neonates with RDS have the highest case fatality rate at 52.5% [3, 4]. The incidence of RDS increases with the decrease of gestation age and is higher in babies less than 30 weeks of gestation age [1].

The use of antenatal steroids to improve pulmonary maturity has been shown to help improve the outcome of preterm babies with RDS [5] but their use is limited to situations where preterm deliveries can be anticipated and steroids started days prior to delivery. To treat preterm babies presenting with RDS effectively, exogenous surfactant and/or mechanical ventilation requires great expertise, equipment plus sustainability. However, these are of limited availability in low and middle income countries [6]. In our setting, we don’t have a neonatal intensive care unit so babies presenting with respiratory distress are treated with oxygen therapy until the symptoms resolve. This may contribute to prolonged hospital stay, predisposing these babies to infection, poor weight gain and risk of developing retinopathy of prematurity which has a 9% incidence rate amongst very low birth weight babies in our setting [7].

An alternative treatment, bubble Continuous Positive Airway Pressure (bCPAP), has been reported to be cheaper, simpler and more accessible to treat RDS [6]. bCPAP is generated by exhalation against a constant opening pressure that produces positive end-expiratory pressure. This in-turn helps in maintaining lung volume at the end of expiration, preventing atelectasis, improving oxygenation, reducing respiratory fatigue and eventually preventing respiratory failure [8, 9]. The use of bCPAP in our setting may help us accelerate the achievement of the Sustainable development goal (SDG) number 3 by reducing the morbidity and mortality in preterm babies presenting with RDS symptoms [10].

Majority of the bCPAP studies have been conducted in developed countries comparing bCPAP to mechanical ventilation and surfactant use in the treatment of RDS in preterm babies [11–13]. The few studies conducted in low and middle income countries have shown a varying effect of bCPAP on survival of preterm babies with RDS ranging from 42% to 85% [6 –16].

Lack of knowledge, equipment and varying results of the effectiveness of bCPAP in studies conducted in low and middle income countries may be contributing to the slow uptake of bCPAP as we do not know whether or not it will favor the survival of preterms in such setups. We aimed to implement and determine the efficacy of bCPAP and its immediate outcomes compared to oxygen therapy in preterm babies presenting with at Kilimanjaro Christian Medical Center (KCMC) Moshi, Tanzania.

## Methodology

### Study design and setting

This was a hospital based randomized controlled trial conducted in the neonatal care unit (NCU) at Kilimanjaro Christian Medical Centre (KCMC) in Northern Tanzania from December 2016 to May 2017. KCMC is a tertiary and referral hospital for the four regions in the Northern part of the country. The NCU at KCMC receives high risk babies delivered within the hospital, at home and referrals from other health facilities. The NCU has a bed capacity of 62 and has no neonatal intensive care unit. The neonatal care unit has 3 nursery rooms; one for preterm babies, one for term babies with non-infectious conditions like birth asphyxia and the third room is reserved for babies with either neonatal sepsis or congenital anomalies. Kangaroo Mother Care is encouraged in the unit and a special room has been allocated for that purpose. Two other rooms are available for stable babies who are given to their mothers to continue treatment under the supervision of the unit nurses, pending discharge when fully recovered. The babies are nursed in locally made baby cots which have low laying bulbs to heat up the cot as well as heaters to keep the babies and room warm. The unit has no mechanical ventilator but had 2 Pumani continuous positive airway pressure machines which were connected to an oxygen concentrator (Airsep, New Life Intensity, 10 LPM) for oxygen supply. Since there is no central oxygen supply, babies received humidified oxygen therapy via nasal prongs or masks from oxygen cylinders.

### Study population

All preterm babies (< 37 weeks of gestation) admitted to the NCU from KCMC labour ward, obstetrics and gynecology theater or referrals presenting with one or more signs of respiratory distress i.e tachypnea (>60breaths/min), chest retraction, nasal flaring, grunting or cyanosis were recruited. Preterm babies with congenital malformations (cleft palate and lip, tracheal esophageal fistula and diaphragmatic hernia) and birthweight less than 1kg were excluded since the Pumani bCPAP machine is to be used on babies with body weight of 1-10kg. Babies whose mothers refused to give consent were also excluded.

### Sampling technique and sample size estimation

Eligible preterm babies who met the inclusion criteria were randomly allocated to either receive bCPAP treatment or oxygen therapy using allocation concealment with brown envelopes picked by the parents or guardian. A 27% survival difference between the two arms, bCPAP and oxygen therapy, was considered to have clinical importance based on the Malawi study which showed a 71% survival in bCPAP and 44% in the oxygen therapy arm [16]. In-order to obtain our sample size we used the 27% survival difference in both arms with the level of significance of 5%, power of 80% and type test was 2 tailed.

Formula used to calculate the sample size:

$$\text{n}={\left(\text{Z}\alpha /2+\text{Z}\beta \right)}^{2}*((\text{p}1\left(1-\text{p}1\right)+\left(\text{p}2\left(1-\text{p}2\right)\right)/{\left(\text{p}1-\text{p}2\right)}^{2}$$

p1 = survival on bCPAP (0.71), p2 = survival on oxygen therapy (0.44), p1 − p2 = 0.27, Zα/2 = 1.96, Zβ = 0.84. The estimated final sample size for this study was 110 participants after adjusting for 10% potential loss due to data collection errors.

### Data collection tools

A questionnaire was used to collect the socio-demographic data and clinical characteristics while a clinical assessment form was used to record vitals and monitor progress of the babies. The Finnistrom score chart was used to assess and determine the gestation age of the babies. The Silverman Anderson Severity Score Chart was used to grade the severity of respiratory distress and a score of ≥6 was used to initiate babies on bCPAP treatment while a score of ≤3 was used to wean the babies off bCPAP. Weaning babies off bCPAP was performed as per the Pumani bCPAP guidelines for clinicians.

### Data collection method

Upon receiving a preterm baby admitted at the NCU and presenting with signs of respiratory distress, a clinical assessment was done taking the vital signs (Respiratory rate, heart rate, temperature, and oxygen saturation), birth weight and assessment of gestation age by the Finnistrom maturity score chart. Severity of respiratory distress was assessed using the Silverman Anderson Score Chart and a physical examination was done to rule out any congenital anomalies like cleft lip and palate. If deemed eligible an informed consent was then sought from the parent or guardian after a detailed explanation of the study by the principle investigator. Thereafter an interview was conducted to obtain the demographic data from parents who gave consent. Parents were then asked to randomly pick a brown envelope in which the mode of treatment the baby would receive was determined and started by the principle investigator. Babies randomized to receive bCPAP were put on bCPAP (Rice 360◦c low cost Pumani bCPAP device) consisting of 3 components: (i) an adjustable flow generator with two pumps used to deliver continuous flow of room air. An oxygen concentrator (Airsep, New Life Intensity, 10 LPM) connected to the device enabling the device to deliver a mixture of pressurized air and oxygen at flow rates ranging from 0-10L/min; (ii) a pressure regulated delivery system determined by the depth of a tube submerged in a water bottle delivering pressures ranging from 5-8cm H_2_O; (iii) the patient nasal interface tubing secured to a stockinette hat with safety pins and elastic bans [15]. The starting bCPAP PEEP in this study was 6cm of water, this was increased by 1cm if the babies`oxygen saturations and respiratory distress were not improving. The maximum bCPAP PEEP in this study was 8cm of water. Babies randomized to the oxygen arm, received humidified oxygen from an oxygen cylinder via nasal prongs.

The babies were examined every 4 to 6 hours to monitor their progress and any treatment complications like pneumothorax, nasal bleeding, injury to the skin, nose or eye, aspiration pneumonia, vomiting and any other complications that would be noted whilst the patient was receiving treatment. Monitoring was done clinically taking the respiratory rate, heart rate, temperature, oxygen saturation and scoring the severity of the respiratory distress. Intermittent monitoring of oxygen saturations was done using pulse oximeters and saturations were kept between 90–96%. Measurement of arterial blood gases was not performed for the study participants.

Normal saline drops were instilled in the nostrils 2–4 hourly to reduce mucosal drying in the babies receiving bCPAP since the Pumani bCPAP machines don’t provide heated or humidified air [15]. Nasal suctioning was performed 2–3 times a day or as per need as nostrils or nasal prongs would get blocked with mucous.

Babies were weaned off bCPAP or oxygen therapy when the signs of respiratory distress resolved and continued observation was done until discharge. Weaning off bCPAP was done if the baby had received bCPAP for at least 24hrs, respiratory rate less than 60 breaths/min for at least 6hrs, oxygen saturations consistently > 90% for at least 6hrs, without significant grunting, recessions, nasal flaring, apnea or bradycardia for at least 6hrs and a Silverman Anderson score of less than ≤3 for at least 6 hours. bCPAP PEEP was then reduced by 1cm of water every 6 hours until 5cm of water was reached. Once 5 cm of water was reached, oxygen flow was reduced by 0.5L/min every 6 hours until 1 L/min was reached. If the baby remained stable on 1 L/min of oxygen and 5cm of water for another 6 hours, bCPAP was stopped. Once off bCPAP, the babies were put on 2 L/min of humidified oxygen therapy via nasal prongs for continued observation for over a 6–12 hour period making sure the baby didn’t develop respiratory distress. The time of starting bCPAP and oxygen therapy, total duration of therapy, time taken to wean off therapy and treatment complications as mentioned earlier were noted throughout the baby`s stay in the neonatal care unit until discharge. According to the baby`s condition and age, they were fed by IV fluids Dextrose 10%, Dextrose saline or expressed breast milk administered via nasal or oral gastric tube. All our study participants were started on antibiotic treatment (ampicillin and gentamycin or ceftriaxone) covering for sepsis whilst awaiting blood culture or C—reactive protein results. If a baby was randomized to the bCPAP arm and the machine was in use, we planned that the baby would receive oxygen therapy whilst awaiting availability of the machine. However this did not arise.

### Study variables

The outcomes of interest were survival, treatment duration and duration of hospital stay. The independent variables included gestational age, birth weight, sex, respiratory rate, mode of delivery, age of mother and mode of treatment.

### Statistical analysis

Data was entered and analyzed using Statistical Package for Social Sciences (SPSS) version 22. Continuous variables were summarized using means and standard deviation (SD) as well as medians and interquartile ranges while categorical variables were summarized using frequency and proportions. An interim analysis was performed after 6 months of beginning the study due to slow recruitment of study participants at which point the study had recruited approximately 45% of the estimated sample size. Survival analysis was conducted to estimate Kaplan-Meier survival functions between the two treatment groups. The logistic-regression model was used to determine the odds of survival between the two treatment groups. Multivariable Cox proportion hazards regression model was performed to estimate hazard ratios (HR) with 95% confidence intervals to determine the risk of death between the two treatment groups. A significance level with P-value of <0.05 was considered statistically significant.

### Ethical consideration

Ethical clearance was obtained from KCMU-College Research Ethics Committee with the certificate number 851 approved on 24^th^ August 2016. Patient recruitment was from 3rd January 2017 to 19th May 2017. Permission to conduct research was also obtained from the head of Paediatrics and child health department at KCMC. Informed consent was obtained from the parents or guardians of the study participants. Privacy and confidentiality was adhered to by using code numbers instead of participant names. Preterm babies on the control arm and those whose parents didn’t consent received the standard treatment for RDS i.e pure oxygen via nasal prongs from the oxygen cylinders. The trial was retrospectively registered at Clinical Trials.gov Identifier: NCT03620448. Registration was not performed in advance because the authors thought the institute ethical clearance was optimal. Once we were made aware of this policy we registered the study as requested. The authors confirm that all ongoing and related trials for this intervention are registered.

## 4. Results

### 4.1. Characteristics of the study participants

**Fig 1:**
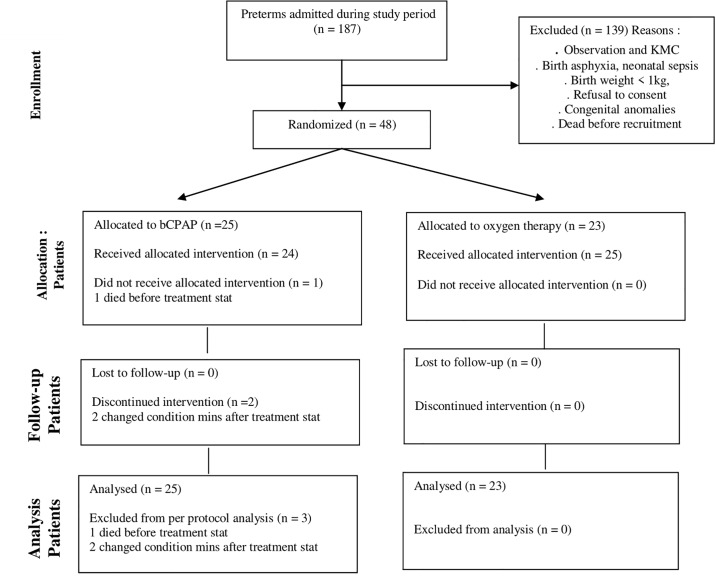
Flow chart showing randomization and treatment allocation of the study participants.

A total of 824 babies were admitted during the study period from January to May 2017. Of these, 187 babies were preterm babies and 48 participants were recruited, randomized and followed up until discharge. The 139 preterm babies not recruited in the study were admitted for observation and care, kangaroo mother care (KMC), birth asphyxia, neonatal sepsis, congenital anomalies and birth weight < 1kg. Some of the parents refused to consent while other eligible participants died before recruitment. Of the 48 participants, 3 babies were excluded after randomization because one died before commencement of treatment and two changed condition needing resuscitation within minutes of commencing treatment. The remaining 45 participants received either bCPAP (n = 22) or oxygen therapy (n = 23) “Fig 1”. No babies were switched from the oxygen arm to the bCPAP arm. At no point was a baby randomized to the treatment group and the bCPAP machine was not available.

#### 4.1.1. Socio-demographic and clinical characteristics of study participants

The demographic and clinical characteristics of the study participants have been summarized in “Table 1”. There was no difference in the baseline characteristics between the treatment and control group. The overall median gestational age was 33 (range 29–36) weeks. Majority (72.9%) had a gestation age ranging from 32 to <37 weeks and birth weight of 1.0–1.4kgs (60.4%). Less than half (39.6%) of the mothers received antenatal steroids before delivery. The mean (SD) Silverman Anderson respiratory severity score at start of the treatment, after 6 hours and 12 hours of treatment was 7.0 (1.2), 4.0 (2.3) and 3.7 (2.2) on bCPAP treatment and 6.5 (2.4), 3.7 (3.0) and 3.0 (2.6) on oxygen therapy, respectively. The mean (SD) of the oxygen saturations amongst the study participants was 94% (3).

**Table 1:** Social demographic and clinical characteristics of participants (N = 48).

Variable	N (%)	bCPAP n (%)	Oxygen n (%)	p value
***Gestational age (weeks)***				
28 to 31 (very preterm)	13(27.1%)	8 (32%)	5 (22%)	
32 to <37 (late preterm)	35(72.9%)	17 (68%)	18 (78%)	0.523
***Median age (range)***	**33 (29–36)**	**33 (29–36)**	**33 (29–35)**	
***Sex***				
Male	29(60.4%)	13 (52%)	16 (70%)	0.250
Female	19(39.6%)	12 (48%)	7 (30%)	
***Birth weight(kg)***				
***1*.*0***–1.4	29 (60.4%)	15 (60%)	14 (61%)	1.000
1.5–2.5	19 (39.6%)	10 (40%)	9 (39%)	
***Median weight (range)***	**1.4 (1–2.3)**	**1.4(1–2.3)**	**1.4(1–1.8)**	
***Place of delivery***				0.653*
KCMC	31 (64.6%)	17 (68%)	14 (61%)	
Other hospitals	16 (33.3%)	8 (32%)	8 (33%)	
Home	1 (2.1%)	0 (0.0%)	1 (4%)	
***Mode of delivery***				
Vaginal delivery	30 (62.5%)	16 (64%)	14 (61%)	1.000
Cesarean section	18 (37.5%)	9 (36%)	9 (39%)	
***Age of mother***				
Mean age (SD)	**28.5 (6.0)**	**27.6 (5.9)**	**29.4 (6.2)**	0.309
***Antenatal steroids***				
Yes	19 (39.6%)	10 (40%)	9 (39%)	1.000
No	29 (60.4%)	15 (60%)	14 (61%)	
***Antenatal steroid doses received***				0.839*
0	29 (60.4%)	15 (60%)	14 (61%)	
1	6 (12.5%)	4 (16%)	2 (9%)	
2	8 (16.7%)	3 (12%)	5 (22%)	
3	1 (2.1%)	1 (4%)	0 (0%)	
4	4 (8.3%)	2 (8%)	2 (9%)	

Value *- Fisher`s Exact Test

A total of 20 deaths were observed and majority (50%) of the deaths occurred in the first 24hrs (10/20) of commencing treatment with 6 deaths occurring in the oxygen arm and 4 in the bCPAP arm. The remaining deaths occurred at 36 to <48 hours (oxygen 2 vs bCPAP 2), 48 to <72 hours (bCPAP 1), >72hours (oxygen 3 vs bCPAP 2).

### 4.2. Primary outcomes

#### 4.2.1. Survival to discharge between the bCPAP arm and oxygen therapy arm

**Fig 2:**
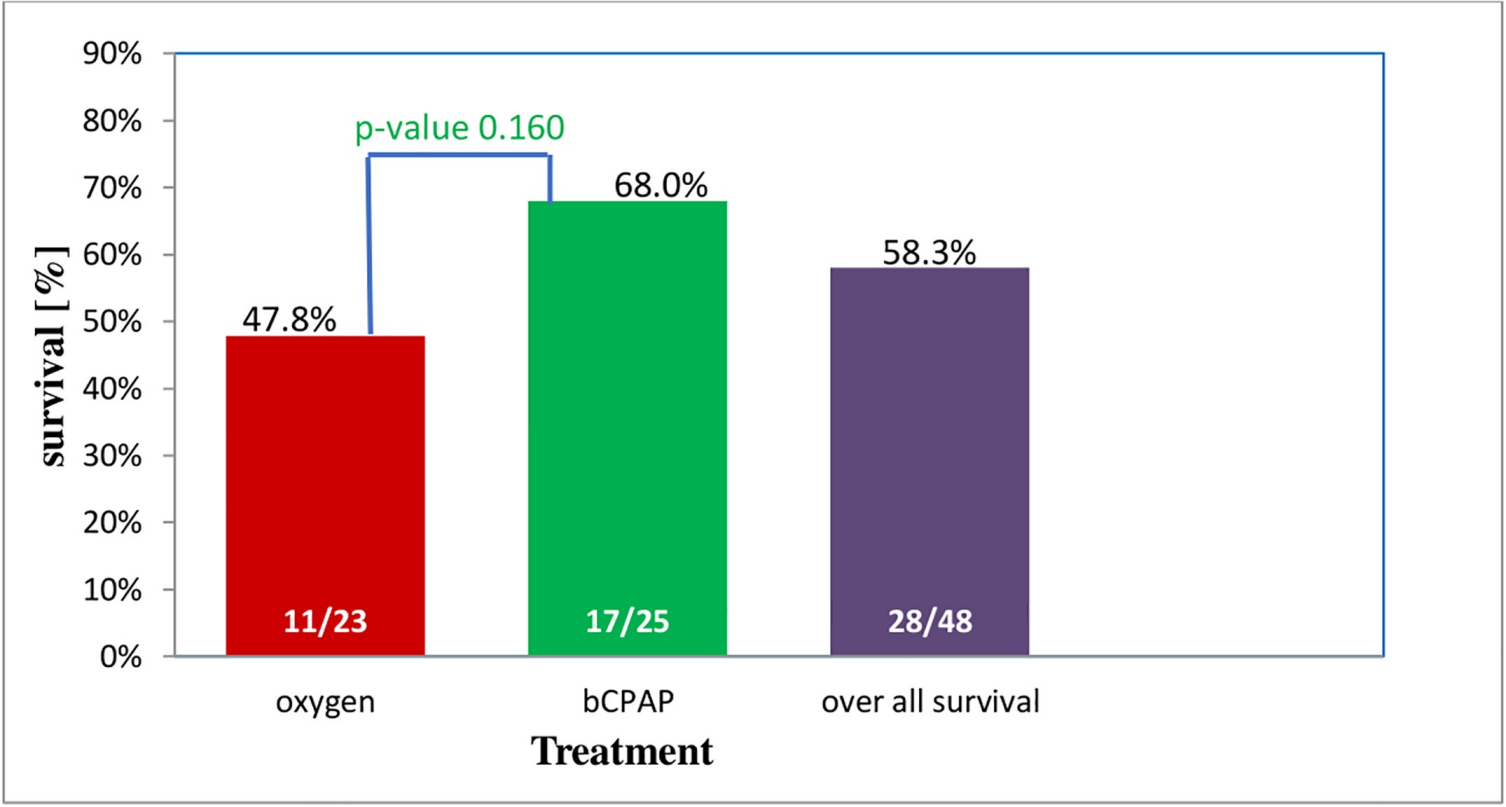
Survival for preterm babies with RDS receiving bCPAP and oxygen (N = 48).

The over-all survival for the 48 participants was 58.3%. Babies treated with bCPAP had a higher survival to discharge compared to their counterparts who were treated with oxygen therapy (68% vs 47.8%), “Fig 2”. This corresponds to 2 fold higher odds of survival as compared to the reference group (crude OR 2.3, 95% CI 0.72–7.49). Babies who adhered to the treatment protocol (N = 45) and received bCPAP also had higher survival to discharge compared to their counterparts (77.2% vs 47.8%) with corresponding odds (crude OR 3.7, 95% CI 1.02–13.47). The overall survival of the babies who adhered to the treatment protocol was 62.2%.

**Fig 3:**
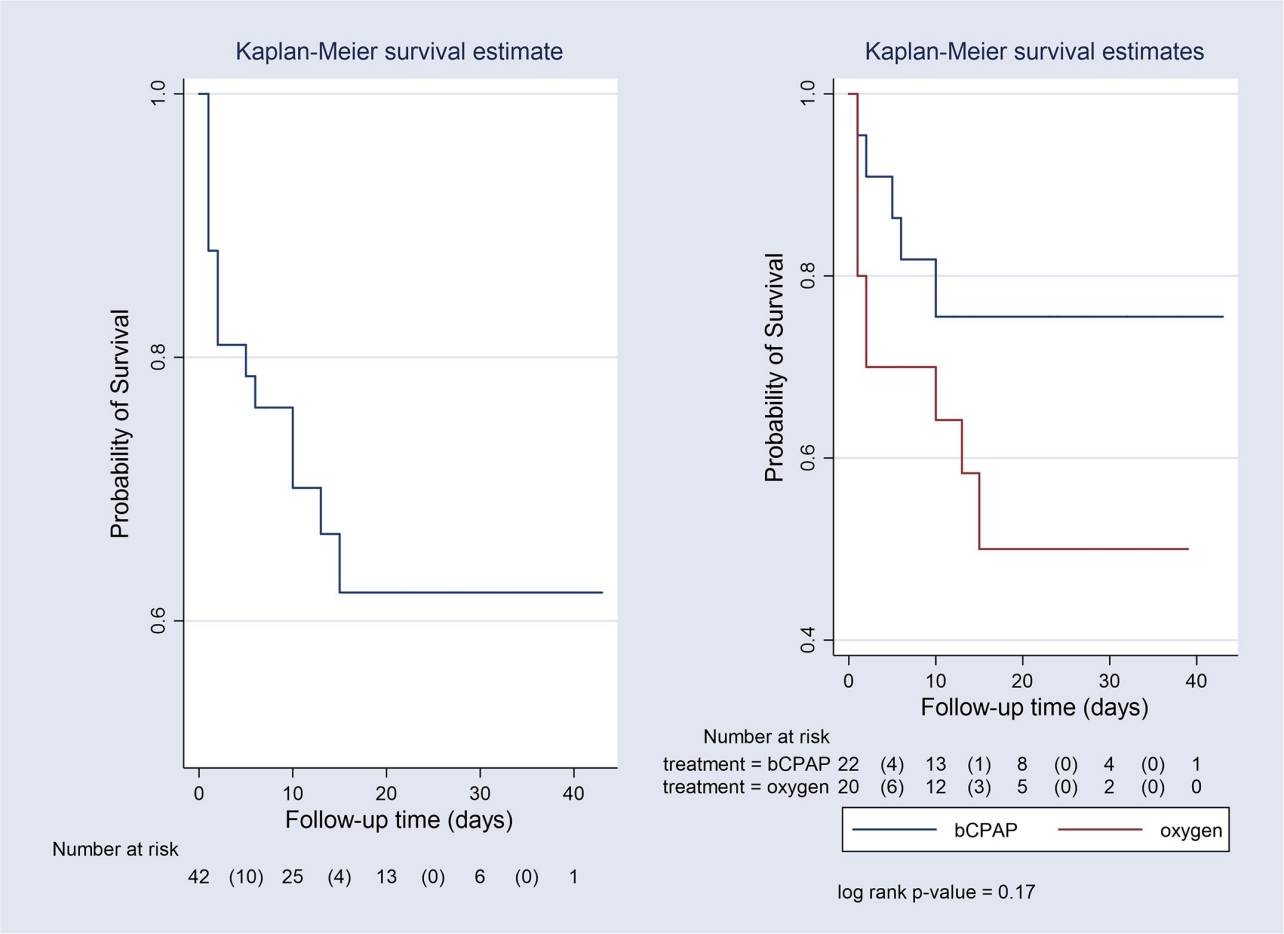
Kaplan-Meier survival estimates by treatment arm.

We compared the difference in survival between the two treatment groups using the Kaplan Meier survival curves while taking into account the censored observations. Of the 48 participants recruited in the study, 6 babies where censored and 42 participants where followed up to discharge. Babies who received bCPAP had a higher survival to discharge compared to babies who received oxygen therapy although this difference was not statistically significant (log rank, P-value 0.17) “Fig 3”.

The findings from the multivariable Cox proportional hazard analysis are shown in “Table 2”. Babies treated with bCPAP had 52% lower risk of death (crude HR 0.48, 95% CI = 0.16–1.43) compared to their counterparts treated with oxygen therapy but this difference didn`t reach statistical significance. Babies with gestational age above 32weeks and birth weight above 1.5kg also showed a lower risk of death though didn’t show statistical significance. Babies whose mothers didn’t receive maternal antenatal steroid treatment and delivered outside KCMC experienced a higher risk of death. Delivery outside KCMC in particular was associated with a 4 fold increase in risk of death (crude HR 4.11, 95% CI = 1.37–12.30).

**Table 2:** Crude hazard ratios for death.

Variable	CHR*(95% CI)	p-value
**Treatment**		
Oxygen	1	
bCPAP	0.48(0.16–1.43)	0.187
**Gestational age (weeks)**		
29–31	1	
32-<37	0.42(0.15–1.22)	0.111
**Sex of the child**		
Male	1	
Female	0.83(0.28–2.47)	0.734
**Birth weight**		
1.0–1.4	1	
1.5–2.5	0.83(0.26–2.73)	0.764
**Place of delivery**		
KCMC	1	
Other hospital	4.11(1.37–12.30)	0.011
**Mode of delivery**		
SVD	1	
Cesarean section	0.43(0.12–1.55)	0.199
**Corticosteroid given to mother**		
Yes	1	
No	2.90(0.81–10.42)	0.102

* Crude hazard ratio

### 4.3. Secondary outcomes

#### 4.3.1. Duration of treatment and hospital stay

The median treatment duration of oxygen therapy was 2 (range 0–16) days and 2 (range 0–5) days for bCPAP respectively. Among the babies who survived to discharge (n = 28), there was no significant difference in the median duration of hospital stay between the bCPAP arm and oxygen arm [14 (range 7–43) days vs 15 (range 6–39) days] “Table 3”.

**Table 3:** Secondary outcomes.

Outcome	bCPAP	Oxygen	P-valve
All eligible participants N = 48	n = 25 median (range)	n = 23 median (range)	
Treatment duration (days)	2(0–5)	2(0–16)	0.543
Hospital stay (days)	10(0–43)	11(0–39)	0.571
**Survivors N = 28**	**n = 17**	**n = 11**	
Treatment duration (days)	2(1–5)	2(1–16)	0.444
Hospital stay (days)	14(7–43)	15(6–39)	1.000

#### 4.3.2. Treatment complications

The babies on the bCPAP arm experienced minimal nasal bleeding (10/25) while vomiting and nasal bleeding was observed in one baby on the oxygen arm.

## Discussion

This study is first of its kind in Northern Tanzania conducted at a tertiary hospital without neonatal intensive care services. Although survival in the bCPAP group was improved by one-third we were not able to show a significant difference with babies receiving oxygen therapy. The median treatment duration between the two groups was found to be similar. The duration of hospital stay amongst the babies that survived to discharge in our study was shorter in the bCPAP group as compared to the oxygen group although not statistically significant. Minor treatment adverse effects such as nasal bleeding were mainly observed with bCPAP treatment when compared to oxygen therapy. Major complications like pneumothorax were not observed among the study participants.

The findings from our study do not contradict other studies in low and middle income countries that showed great improvement of survival to discharge in preterm babies when treated with bCPAP [16–20]. Pieper and colleagues conducted a small non-randomized study in South Africa demonstrating increased survival rates of extremely immature babies with moderate to severe respiratory distress treated with nasal CPAP in the absence of surfactant replacement therapy and neonatal intensive care [17].

The use of the Silverman Anderson Severity Score Chart together with the respiratory distress symptoms not only helped us in early identification and commencement of treatment but also helped in monitoring the progress of these babies on treatment and determined which babies were fit for weaning off bCPAP. McAdams and colleagues in Uganda reported a survival to discharge rate of 61% amongst babies treated with bCPAP and also noted the effectiveness of the Silverman Anderson Severity Score as a reliable tool in treating and monitoring babies on bCPAP. It was also noted that the assessment skill of the Silverman Anderson Severity Score was easily grasped and retained over time by both the nurses and doctors [20]. Similarly in Rwanda, Nahimana et al. recommended that in a setting with a high turnover of staff the use of standard clinical charts like the Silverman Anderson Severity Score Chart would be helpful in correct identification of eligible newborns with respiratory distress and standardization of care [14]. In the present study, we observed a similar median duration of treatment with bCPAP (2 (range 0–5) days) and oxygen therapy (2 (range 0–16) days). Our results do not contradict Saha et al, who reported a reduced treatment duration with bCPAP as compared to oxygen therapy (3.69±1.55 vs 7.67±2.76) [19]. This was in contrast to the study in Malawi were the authors reported a longer mean treatment duration of 6.9(SD5.37) days on the bCPAP arm compared to the oxygen therapy arm 4.0days (SD4.37) days. This difference could be explained by the movement of babies from the control group to the bCPAP arm when the bCPAP machine was available increasing the number of treatment hours on the bCPAP arm [16].

Survivors in the oxygen arm were noted to have a slightly longer duration of hospital stay compared to their counterparts in the bCPAP group although the difference is one day and not statistically significant. Similarly a study done in Bangladesh showed the mean duration of hospital stay was longer in babies receiving oxygen therapy (12.67±11.75 days) compared to the bCPAP group which was 8.74±3.72 days [19]. This is in contrast to the study done in Malawi where they observed treatment with oxygen therapy to have a shorter duration of hospital stay as compared to treatment with bCPAP [16]. A study in Australia reported a shorter hospital stay in both bCPAP and oxygen treatment groups as 6.0 vs 5.0 days respectively [21]. In this study they used surfactant which is another effective mode of treatment for RDS hence reducing RDS morbidity. We didn’t observe serious effects like pneumothorax as reported in the Australian study [21]. The Australian study had a larger sample size than our study and also recruited extreme low birth weight babies who might not have tolerated the bCPAP machine. Similar findings were reported by Mathai et al where 2 of the 3 babies with body weight less than 1kg failed bCPAP [22]. The starting bCPAP PEEP in both studies was 6cm of water which was similar to our study.

Our study also observed an increased risk of deaths among preterm babies who were born out of our facility and those whose mothers didn’t receive antenatal steroids. This has been reported in other studies showing poor outcomes in such babies [1–5]. The 3 delays model extrapolated from the 3 delays in maternal mortality could explain the possible factors resulting in the poor outcomes which include delay in decision to seek care, delay in reaching/accessing care and delay in receiving appropriate care [23].

Our study suggests that bCPAP can help reduce the morbidity and mortality among preterm babies with respiratory distress and should be considered as the mainstay of treatment for these babies. This may pave the way for bCPAP roll out on a large scale in our country, improving the management of these babies and reducing the need of referring preterm babies with respiratory distress.

This study was limited to 2 bCPAP machines and also experienced slow recruitment of participants similar to a non-randomized trial in South Africa by Pieper et al,. Since our inclusion criteria did not include any definitive test to diagnose RDS like a chest x-ray, there could have been infants in the study who had other respiratory conditions such as infections or transient tachypnea of the newborn.

## Conclusion

Although there was a 30% difference in survival to discharge, which approximated to the 27% survival that was reported by the Malawi study [16], this study was unable to demonstrate any significant difference between the bCPAP and control group. The most likely reason for this is that we failed to recruit sufficient study participants. We note that because we did not meet our calculated sample size due to limitations of bCPAP machines and slow recruitment of participants, this finding should be treated with caution and could be a chance finding. Nevertheless the findings concur with other studies reviewed by Martin et al, 2014. Our findings justify the need for further adequately powered studies in this or similar settings. Such studies would need to be adequately supported including sufficient bCPAP units to carry out the study in a timely way. No difference was noted in the treatment duration between the treatment arm and oxygen therapy. Again this may be attributed to the small sample size of the study. To determine the long term effects of oxygen therapy like retinopathy of prematurity, we recommend neurological developmental follow-up for these babies to determine chronic complications like retinopathy of prematurity. Although more adverse effects were observed in the bCPAP group, its benefits out way the risks and a risk like nasal bleeding can be prevented by 2 to 4 hourly nasal irrigation with normal saline drops. We recommend that bCPAP be considered the standard treatment for babies with respiratory distress. Staff should be trained on the use of bCPAP and its efficacy. Future research should be done in other groups of neonates and infants presenting with respiratory distress to expound on the effect of bCPAP.

## Supporting information

S1 Fig Silverman Anderson score chart. (DOCX)

S2 Fig Finnistrom score chart. (DOCX)

S3 Fig Setup of bubble-CPAP machine. (DOCX)

S4 Fig Flow chart for weaning a baby off bCPAP. (DOCX)

S1 Checklist (DOC)
