# Supplementary material for: Treatment outcomes of Pumani bubble-CPAP versus oxygen therapy among preterm babies presenting with respiratory distress at a tertiary hospital in Tanzania—Randomised trial
Source: PLoS One. 2020 Jun 30;15(6):e0235031. doi: 10.1371/journal.pone.0235031 (PMC7326169; doi:10.1371/journal.pone.0235031)
Supplement: S1 Fig — (DOCX) [file pone.0235031.s001.docx]

## S1 Fig: Silverman Anderson Score Chart


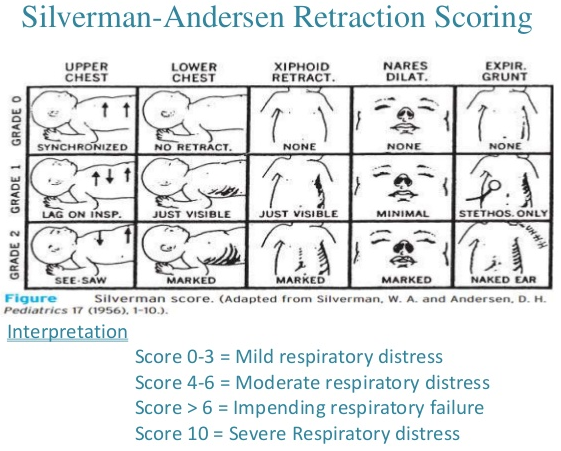


Score >6 impending respiratory failure need for respiratory support – CPAP or Ventilation

Score >6 impending respiratory failure need for respiratory support – CPAP or Ventilation
