# Supplementary material for: Treatment outcomes of Pumani bubble-CPAP versus oxygen therapy among preterm babies presenting with respiratory distress at a tertiary hospital in Tanzania—Randomised trial
Source: PLoS One. 2020 Jun 30;15(6):e0235031. doi: 10.1371/journal.pone.0235031 (PMC7326169; doi:10.1371/journal.pone.0235031)
Supplement: S2 Fig — (DOCX) [file pone.0235031.s002.docx]

## S2 Fig: Finnistrom Score Chart


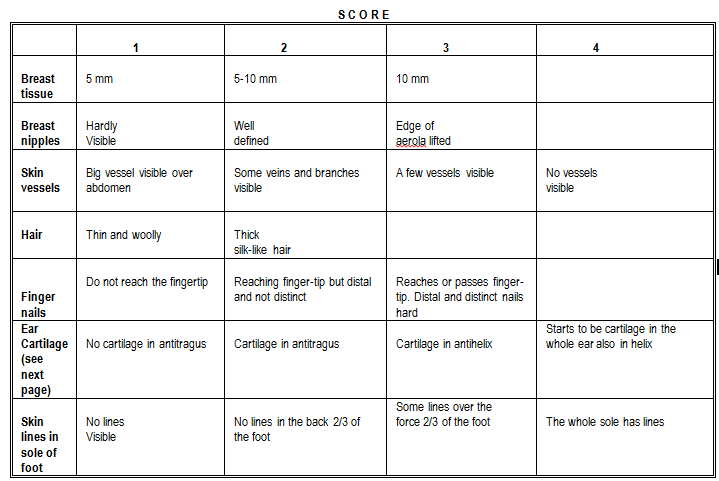


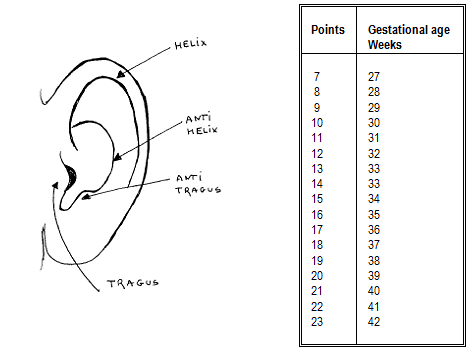


Figure showing the Finnistrom maturity score chart for assessing gestation of newborn babies born within 24hours. Adopted from KCMC Blue book 7^th^ edition.
