# Supplementary material for: Treatment outcomes of Pumani bubble-CPAP versus oxygen therapy among preterm babies presenting with respiratory distress at a tertiary hospital in Tanzania—Randomised trial
Source: PLoS One. 2020 Jun 30;15(6):e0235031. doi: 10.1371/journal.pone.0235031 (PMC7326169; doi:10.1371/journal.pone.0235031)
Supplement: S3 Fig — (DOCX) [file pone.0235031.s003.docx]

## S3 Fig: Setup of Bubble-CPAP Machine


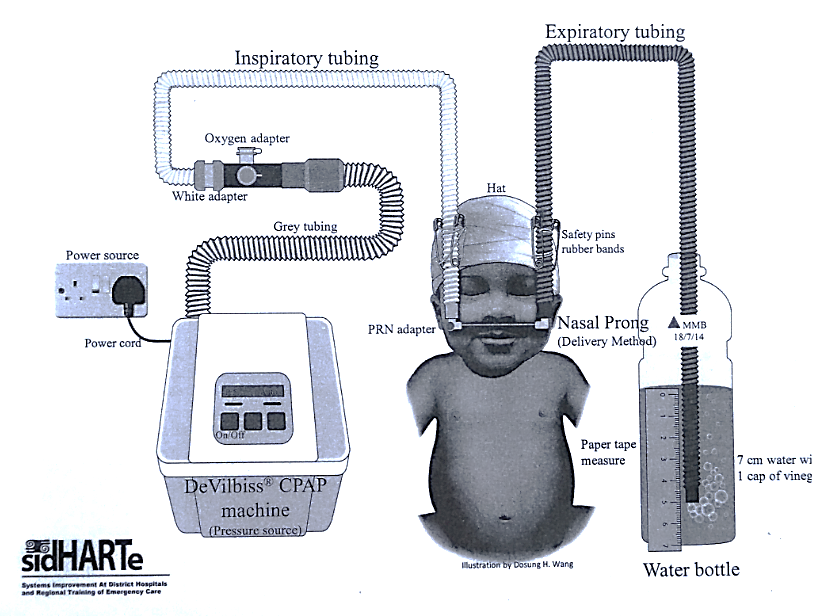


Setup of baby receiving bCPAP, adopted from suppliers of the pumani bCPAP machine in Kenya.
