# Supplementary material for: Treatment outcomes of Pumani bubble-CPAP versus oxygen therapy among preterm babies presenting with respiratory distress at a tertiary hospital in Tanzania—Randomised trial
Source: PLoS One. 2020 Jun 30;15(6):e0235031. doi: 10.1371/journal.pone.0235031 (PMC7326169; doi:10.1371/journal.pone.0235031)
Supplement: S4 Fig — (DOCX) [file pone.0235031.s004.docx]

## S4 Fig: Flow chart for weaning a baby off bCPAP


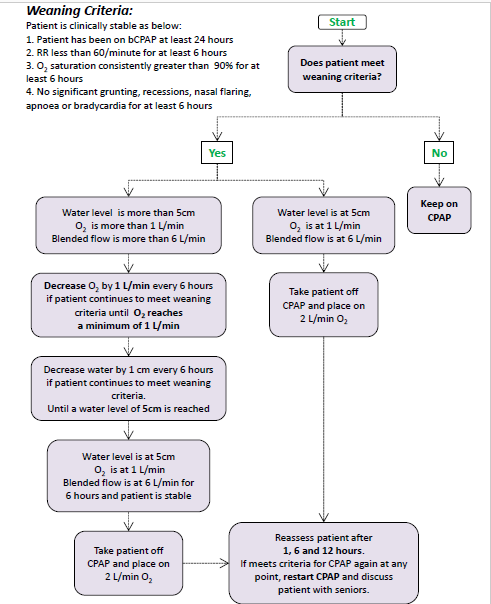


A flowchart showing how to weaning a patient from CPAP, adapted from Rice 360^◦^C Institute of Global Health.
